# Changes of inflammatory and oxidative stress biomarkers in dogs with different stages of heart failure

**DOI:** 10.1186/s12917-020-02650-7

**Published:** 2020-11-10

**Authors:** Camila Peres Rubio, Ahmet Saril, Meriç Kocaturk, Ryou Tanaka, Jorgen Koch, Jose Joaquin Ceron, Zeki Yilmaz

**Affiliations:** 1 Interdisciplinary Laboratory of Clinical Pathology, Interlab-UMU, University of Murcia, 30100 Murcia, Spain; 2 Department of Internal Medicine, Faculty of Veterinary Medicine, Bursa Uludag University, 16059 Bursa, Turkey; 3 Department of Veterinary Surgery, Faculty of Veterinary Medicine, Tokyo University of Agriculture and Technology, Tokyo, 183-8509 Japan; 4 Department of Veterinary Clinical Sciences, Faculty of Health and Medical Sciences, University of Copenhagen, Frederiksberg - Copenhagen, Denmark

**Keywords:** Cytokines, Chemokines, Inflammation, Oxidative stress, Heart failure, Dogs

## Abstract

**Background:**

Heart failure (HF) is associated with changes in inflammatory and oxidative stress biomarkers. This study aimed to evaluate the changes of a panel of inflammatory and oxidative stress biomarkers in dogs with different stages of HF and its relation with the severity of the disease and echocardiographic changes. A total of 29 dogs with HF as a result of myxomatous mitral valve degeneration or dilated cardiomyopathy were included and classified as stage-A (healthy), B (asymptomatic dogs), C (symptomatic dogs) and D (dogs with end-stage HF) according to the ACVIM staging system. In these dogs an ecnhocardiographic examination was performed and cytokines, and inflammatory and oxidative stress markers were evaluated in serum.

**Results:**

KC-like was significantly increased in dogs of stage-C (*P* < 0.01) and -D (*P* < 0.05) compared with stage-A and -B. Stage-D dogs showed significantly higher serum CRP and Hp (*P* < 0.05) but lower serum antioxidant capacity (PON1, TEAC, CUPRAC, and thiol) compared to stage-A and -B (*P* < 0.05). After the treatment, serum levels of CRP, Hp and KC-like decreased and serum antioxidant levels increased compared to their pre-treatment values. Left ventricular dimension and LA/Ao ratio correlated positively with CRP, MCP-1, and KC-like but negatively with PON1, GM-CSF, IL-7 and antioxidant biomarkers (*P* < 0.01).

**Conclusion:**

Our results showed that dogs with advanced HF show increases in positive acute-phase proteins and selected inflammatory cytokines such as KC-like, and decreases in antioxidant biomarkers, indicating that inflammation and oxidative stress act as collaborative partners in the pathogenesis of HF. Some of these biomarkers of inflammation and oxidative stress could have the potential to be biomarkers to monitor the severity of the disease and the effect of treatment.

## Background

Chronic heart failure (CHF) is a progressive clinical syndrome and characterized by exercise intolerance, dyspnea, coughing, lethargy, abdominal distension (ascites), and decrease in the quality and duration of life due to impaired cardiac and pulmonary functions [1]. Myxomatous mitral valve degeneration (MMVD) and dilated cardiomyopathy (DCM) are the most common naturally occurring heart diseases eventually resulting in CHF in dogs [2, 3] and humans [4].

MMVD is characterized by progressive myxomatous degeneration of mitral valve leaflets leading to mitral regurgitation and left-sided cardiac remodeling with a general preserved systolic function [2]. Dilated cardiomyopathy (DCM) has recently emerged as having a genetic basis primarily in large breeds and is characterized by cardiomegaly with predominantly impaired left ventricular systolic function. Ascites is due to right-sided CHF and it is often associated with biventricular failure in giant breeds with DCM [2, 4, 5].

Although both of them are commonly considered non-inflammatory conditions [6, 7], studies have found increased circulating inflammatory cytokines in dogs and humans with CHF due to MMVD [8, 9] and DCM [7, 10]. Increased expression and release of inflammatory cytokines such as tumour necrosis factor (TNF-α), as well as serum C-reactive protein (CRP), have been described in humans [11, 12] and dogs with CHF [13]. Increased levels of monocyte chemoattractant protein 1 (MCP-1) and decreased levels of interleukins have also been found in dogs with CHF [8], but there is no data available on how CRP or other inflammatory biomarkers can change in severe cases [6]. Although increased levels of oxidative stress biomarkers are associated with cardiovascular diseases in dogs [14–16] and humans [17, 18], there are no studies describing oxidative stress biomarkers such as total antioxidant capacity and thiol, and their relationships with inflammatory biomarkers and echocardiographic variables among the different stages of CHF.

The objectives of this study were to evaluate a panel of serum inflammatory and oxidative stress biomarkers in dogs with different stages of heart failure classified according to the American College of Veterinary Internal Medicine (ACVIM) guidelines [3], and to study the correlation between these biomarkers and echocardiographic variables. Namely, a cytokine panel including 13 cytokines, inflammatory biomarkers such as ferritin, CRP, haptoglobin (Hp), paraoxonase 1 (PON1) and butyrylcholinesterase (BChE) and oxidative stress markers such as total antioxidant capacity (cupric reducing antioxidant capacity - CUPRAC, and trolox equivalent antioxidant capacity - TEAC) and total thiol were evaluated. Furthermore, we sought to evaluate the changes of these biomarkers after treatment in dogs with severe CHF.

## Results

### Animals

The groups of the study were integrated by different breeds. Stage A group included 3 Labrador, 2 Border Collie, 1 Samoyed, 1 Cavalier King Charles Spaniel, and 1 Anatolian shepherd. Stage B2 group included 2 Golden Retrievers, 1 Cocker Spaniel, 1 Kopay, 1 Jack Russell, and 1 Shih Tzu. Stage C group included 2 Anatolian shepherds, 3 Pekingeses, 1 Shih Tzu, 1 Cavalier King Charles Spaniel, 1 Pincher, and 3 Terriers. Stage D group included 3 Anatolian shepherds, 2 Cocker Spaniels, 1 Pit Bull, 1 German shepherd, and 1 mix breed.

### Clinical data

There was a statistically significant difference in ages between dogs with stage A and C (*P* < 0.05) as well as stage A and D (*P* < 0.01) (Table 1). Heart and respiratory rates increased in parallel according to the severity of the diseases from stages A to D (data not shown) (*P* < 0.01).

**Table 1:** Selected clinical, hematological and echocardiographic parameters in dogs with different stages (Stages A, B2, C, and D) of heart failure. Mean ± SEM

Parameters	Stage A (*n* = 8)	Stage B2 (*n* = 6)	Stage C (*n* = 10)	Stage D (*n* = 5)
**Clinical variables**				
Age (years)	3.5 ± 0.3^a^	6.0 ± 1.6^ab^	8.6 ± 1.6 ^b*^	9.1 ± 1.8 ^b**^
Body Weight (Kg)	23.5 ± 3.8 ^a^	19.1 ± 4.7 ^a^	16.0 ± 5.6 ^a^	34.1 ± 8.5 ^a^
Gender (M/F)	2 / 6	2 / 4	6 / 4	3 / 2
**Haematological variables**				
WBC (×10^3^/mm^3^)	11.5 ± 1.1^a^	10.1 ± 1.4^a^	18.4 ± 2.5^b#**^	18.7 ± 1.5^b#**^
Neu (× 10^3^/mm^3^)	7.9 ± 0.8 ^a^	7.3 ± 0.9 ^a^	14.4 ± 2.2 ^b**^	15.7 ± 1.3^b**^
cTnI ng/mL	0.03 ± 0.04 ^a^	0.03 ± 0.03 ^a^	1.80 ± 1.39 ^b***^	5.30 ± 2.01 ^c***^
**Echocardiographic variables**				
RVd (cm)	0.62 ± 0.09 ^a^	0.96 ± 0.11 ^a^	0.97 ± 0.33 ^a^	1.36 ± 0.35 ^a^
IVSd (cm)	0.9 ± 0.1 ^a^	1.1 ± 0.1 ^a^	0.7 ± 0.0 ^a^	1.0 ± 0.1 ^a^
IVSs (cm)	1.1 ± 0.1 ^a^	1.3 ± 0.1 ^a^	0.9 ± 0.0 ^a^	1.2 ± 0.1 ^a^
LVd (cm)	3.2 ± 0.2 ^a^	3.0 ± 0.5 ^a^	3.9 ± 0.4 ^a^	5.7 ± 2.0 ^b#**^
LVs (cm)	2.2 ± 0.2 ^a^	2.1 ± 0.1 ^a^	2.8 ± 0.4 ^a^	4.3 ± 0.8 ^b**#*^
PWd (cm)	1.0 ± 0.1 ^a^	0.9 ± 0.1 ^a^	0.6 ± 0.0 ^b**^	0.9 ± 0.0 ^a^
PWs (cm)	1.2 ± 0.1 ^a^	1.2 ± 0.1 ^a^	0.7 ± 0.0 ^b***^	1.3 ± 0.1 ^a^
LA /Ao	1.1 ± 0.0 ^a^	1.6 ± 0.1 ^b^	2.2 ± 0.1 ^b#***^	2.3 ± 0.1 ^bc#***^
LVIDDn	1.38 ± 0.31^a^	1.72 ± 0.12 ^b**^	1.78 ± 0.18^bc**^	2.03 ± 0.49^c***#***^
MV E/A	1.9 ± 0.21 ^a^	2.2 ± 0.2 ^ab^	2.2 ± 0.1 ^ab^	3.0 ± 0.5 ^b*^
EPSS (cm) (DCM/MMVD)	0.2 ± 0.1^a^	0.4 ± 0.0^a^ (0.7 ± 0.3/0.2 ± 0.1^+^)	0.5 ± 0.1 ^ab^ (1.2 ± 0.3/0.3 ± 0.1^+^)	1.2 ± 0.3 ^b*^ (1.5 ± 0.4/0.4 ± 0.1^+^)
FS (%) (DCM/MMVD)	32 ± 2 ^a^	35 ± 2^a^ (31 ± 3/38 ± 5)	29 ± 2 ^a^ (16 ± 6/33 ± 7^+^)	26.7 ± 5.5 ^a^ (16 ± 7/47 ± 9^+^)

In the same rows, the difference between different letters was statistically significant, but the difference between all values with the same letter was not significant. ^*^ *P* < 0.05; ^**^ *P* < 0.01; ^***^ *P* < 0.001

^#^ Compared with Stage B2

+ Comparison between dilated cardiomyopathy (DCM) and myxomatous mitral valve disease (MMVD) (at least, *P* < 0.05)

### Echocardiographic variables

Table 1 shows the echocardiographic variables of dogs in this study. M-mode measurements showed that LV dimensions at diastole and systole were increased in stage D compared to those of other stages of HF (*P* < 0.01). There were statistically increases in LA/Ao ratio (*P* < 0.01), LVIDDn (*P* < 0.001), and EPSS values (*P* < 0.05) between stage A and B and stage C and D. Mitral valve E/A ratios in stage D were higher than those of other stages of HF (*P* < 0.05). FS values did not differ statistically between the groups (Table 1). EPSS and FS values were presented separately for DCM and MMVD in Table 1. EPSS values in dogs with DCM were higher, whereas FS values were lower than those of dogs with MMVD within and between groups, at least *P* < 0.05.

### Hematologic and serum biochemical results

Regarding the CBC results, stage C and D showed increases (*P* < 0.01) in WBC and neutrophil counts, compared to stages A and B (Table 1). RBC and PLT counts did not differ statistically between groups (data not shown). There was a significant increase in cTnI level in stage C (*P* < 0.01; vs stage A and B) and stage D (*P* < 0.001; vs stage A, B2 and C) (Table 1). There were no statistically significant differences in biochemical parameters studied between the groups (data not shown).

### Inflammatory biomarkers

**Fig. 1:**
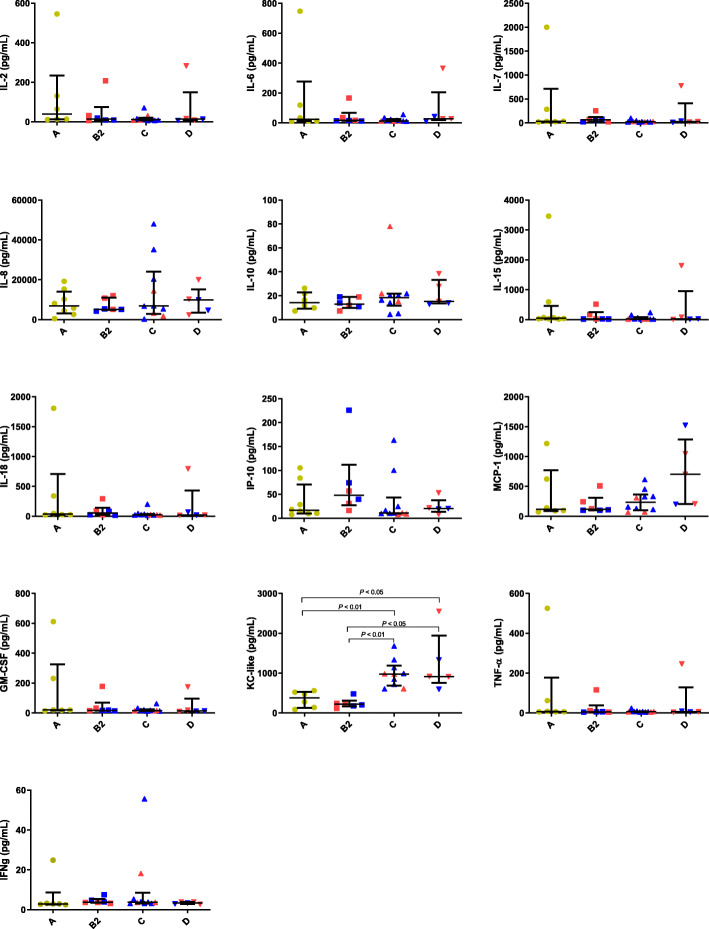
Cytokine results in dogs of different stages of heart failure: Stage A, Stage B2, Stage C and Stage D. IL, interleukin; IP-10, interferon gamma-induced protein 10; MCP-1, monocyte chemoattractant protein 1, GM-CSF, granulocyte-macrophage colony-stimulating factor; KC-like, keratinocyte-derived chemokine; TNF-α, tumor necrosis factor–alpha; IFN-γ, interferon–gamma. Red and blue colors represent data from dogs with dilated cardiomyopathy (DCM) and myxomatous mitral valve disease (MMVD), respectively

The cytokines results are shown in Fig. 1. Significant increases in KC-like in dogs of stage C (median/interquartile range: 972/689–1188 pg/mL; *P* < 0.01) and D (median/interquartile range: 917/753–1942 pg/mL; *P* < 0.05) were found when compared with stage A (median/interquartile range: 377/125–531 pg/mL) and B dogs (median/interquartile range: 222/163–309 pg/mL). No significant differences in IL-2, IL-6, IL-7, IL-8, IL-10, IL-15, IL-18, IP-10, MCP-1, GM-CSF, TNF-α and IFN-γ results were observed between the different groups of dogs (*P* > 0.05).

**Fig. 2:**
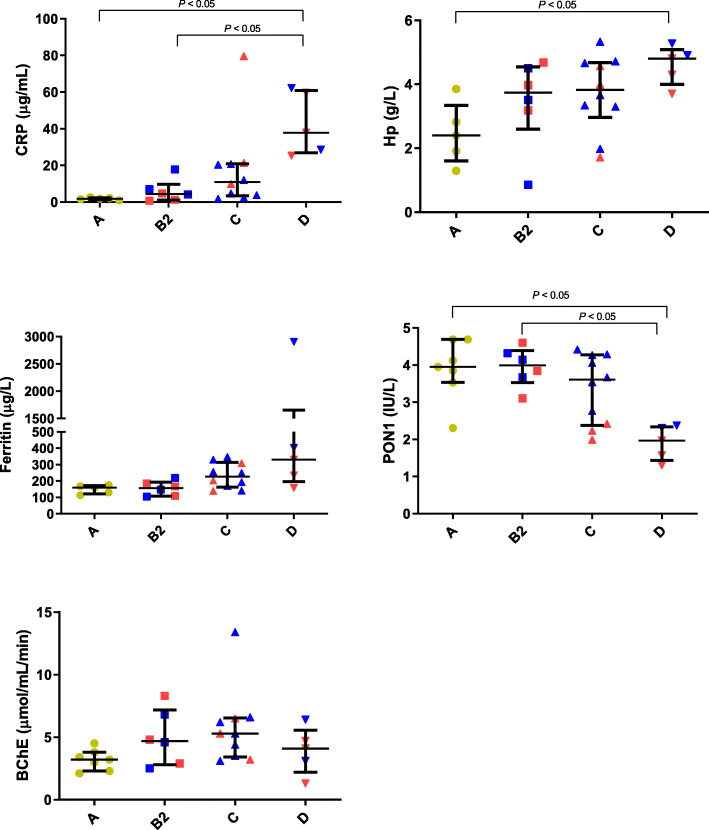
Values of C-reactive protein (CRP), haptoglobin (Hp) and ferritin in dogs with different stages of heart failure: Stage A, Stage B2, Stage C and Stage D. The plots show median, 25th and 75th percentiles. Red and blue colors represent data from dogs with dilated cardiomyopathy (DCM) and myxomatous mitral valve disease (MMVD), respectively

**Fig. 3:**
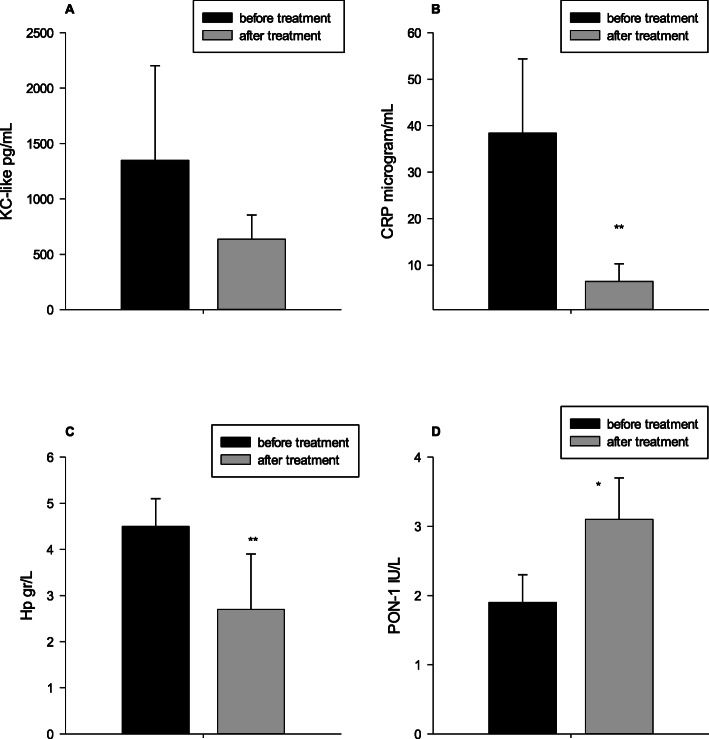
Serum concentrations of keratinocyte chemotactic like (KC-like) (**a**), C-reactive protein (CRP) (**b**), haptoglobin (Hp) (**c**) and paraoxanase-1 (PON-1) (**d**), before (*n* = 5) and 2 weeks after the treatments (*n* = 5) in stage D of chronic heart failure. * *P* < 0.05 ** *P* < 0.01

**Fig. 4:**
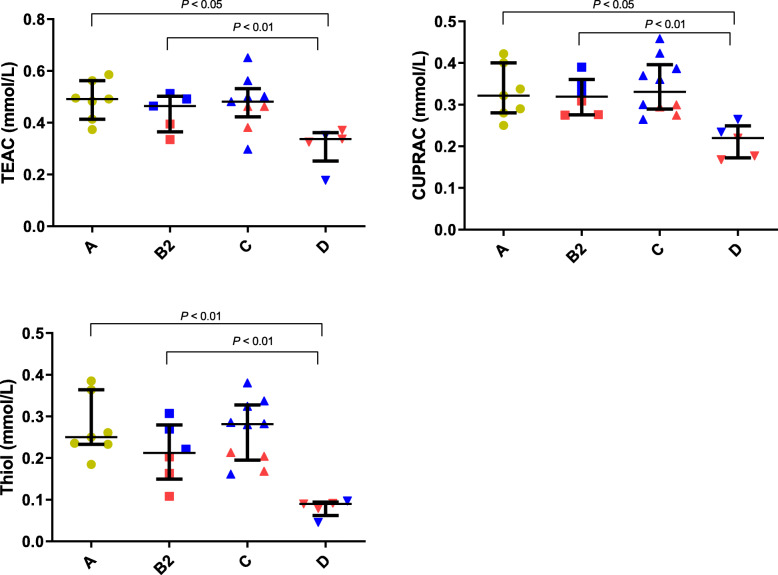
Trolox equivalent antioxidant capacity (TEAC), cupric reducing antioxidant capacity (CUPRAC), and thiol concentrations in serum of dogs with different stages of heart failure: Stage A, Stage B2, Stage C and Stage D. The plots show median, 25th and 75th percentiles. Red and blue colors represent data from dogs with dilated cardiomyopathy (DCM) and myxomatous mitral valve disease (MMVD), respectively

A significantly higher serum CRP concentration (Fig. 2) was observed in the stage D of CHF (median/interquartile range: 37.8/26.9–60.9 μg/mL) compared with stage A (median/interquartile range 2.1/1.5–10.9 μg/mL, *P* < 0.05) and with stage B2 dogs (median/interquartile range 4.3/1.0–9.7 μg/mL, *P* < 0.05). Hp concentrations were significantly higher in dogs with stage D when compared with stage A dogs (median/25th–75th percentiles: 2.4/1.6–3.3 versus 4.8/3.9–5.0 g/L, *P* < 0.05). A significantly lower PON1 activity (Fig. 4) was found in dogs with Stage D of CHF (median/25th–75th percentiles: 1.9/1.4–2.3 IU/L) compared with stage A dogs (median/25th–75th percentiles: 3.9/3.5–4.6 IU/L, *P* < 0.05) and with dogs with stage B2 (median/25th–75th percentiles: 3.9/3.5–4.3 IU/L, *P* < 0.05). Despite that groups were not sub-divided as DCM and MMVD, data from these dogs were coded by different colors (Fig. 4); serum PON1 activity of dogs with DCM (red color) seemed to be lower than that of dogs with MMVD (blue color) within groups in the more severe stages such as stage C and D and between different groups (stage C vs stage A and B2). No significant differences were found in serum ferritin concentrations and BChE activity between stage A dogs and the different groups of dogs with CHF (Fig. 2). KC-like, CRP (*P* < 0.01), Hp (*P* < 0.01) and PON1 (*P* < 0.05) showed significant variations after 2 weeks of the therapy (Fig. 3).

### Oxidative stress markers

**Fig. 5:**
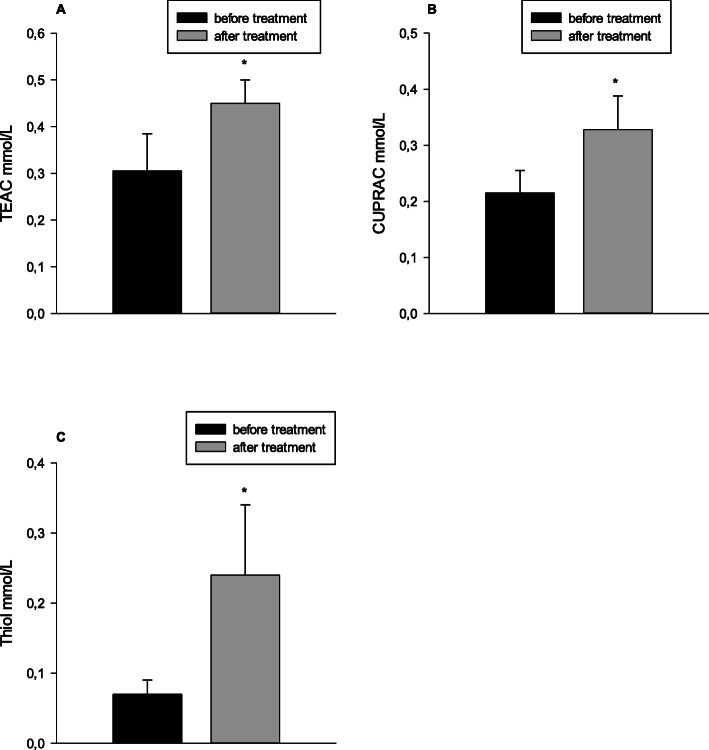
Serum concentrations of trolox equivalent antioxidant capacity (TEAC) (**a**), cupric reducing antioxidant capacity (CUPRAC) (**b**) and thiol (**c**), before (*n* = 5) and 2 weeks after the treatments (*n* = 5) in stage D of chronic heart failure. * *P* < 0.05

Variations in the markers of oxidative stress analyzed in the different groups of dogs are shown in Fig. 4. Dogs with stage D heart failure presented significant lower concentrations of serum TEAC (median/25th–75th percentiles: 0.34/0.25–0.36 mmol/L, *P* < 0.05), CUPRAC (median/25th–75th percentiles: 0.22/0.17–0.25 mmol/L, *P* < 0.05) and thiol (median/25th–75th percentiles: 0.09/0.06–0.09 mmol/L, *P* < 0.05) when compared to stage A dogs (median/25th–75th percentiles: 0.49/0.41–0.56 mmol/L [TEAC]; 0.32/0.28–0.40 mmol/L [CUPRAC]; 0.25/0.22–0.36 mmol/L [thiol]) and with stage B2 dogs (median/25th–75th percentiles: 0.46/0.36–0.50 mmol/L, *P* < 0.01 for TEAC; 0.31/0.27–0.36 mmol/L, *P* < 0.01 for CUPRAC; 0.21/0.14–0.27 mmol/L, *P* < 0.01 for thiol). Concentrations of TEAC, CUPRAC, and thiol increased after 2 weeks of therapy in dogs with stage D of CHF (Fig. 5).

### Correlation study

Table 2 shows all correlations with statistical significance (*P* < 0.05) found between echocardiographic parameters and biomarkers studied. The highest correlation (ρ > 0.70) was observed between LA/Ao and serum cTnI (*P* < 0.001). CRP positively correlated with LVDd (ρ = 0.40; *P* = 0.035), LVIDDn (ρ = 0.41; *P* = 0.037) and LA/Ao (ρ = 0.60; *P* = 0.001), in addition KC-like and MCP-1 correlated positively with LA/Ao (ρ = 0.52; *P* = 0.004) and LVIDDn (ρ = 0.41; *P* = 0.003), respectively. All antioxidant biomarkers were negatively correlated with LVIDDn, being the highest coefficient of correlation with CUPRAC (ρ = − 0.52; *P* = 0.006). There were not statistically significant differences between gender and measured parameters.

**Table 2:** Spearman correlation coefficient between echocardiographic parameters and biomarkers studied of all dogs included in this study. The correlations included in this table are only those that showed statistical significance (*P* < 0.05)

Parameter	Spearman’s rank correlation coefficient						
WBC	cTnI (ρ = 0.57)	LA/Ao (ρ = 0.55)					
NEU	cTnI (ρ = 0.62)	FS (ρ = − 0.36)	LA/Ao (ρ = 0.60)	WBC (ρ = 0.96)			
BUN	cTnI (ρ = 0.40)	WBC (ρ = 0.50)	NEU (ρ = 0.56)				
CRP	cTnI (ρ = 0.39)	LVDd (ρ = 0.40)	LVIDDn (ρ = 0.41)	LA/Ao (ρ = 0.60)	WBC (ρ = 0.56)	NEU (ρ = 0.60)	
Ferritin	cTnI (ρ = 0.51)	LA/Ao (ρ = 0.46)	WBC (ρ = 0.41)	NEU (ρ = 0.46)	CRP (ρ = 0.54)	Hp (ρ = 0.47)	BChE (ρ = 0.43)
PON1	cTnI (ρ = − 0.41)	LVIDDn (ρ = − 0.62)	LVDd/s (ρ = − 0.63/− 0.49)	LA/Ao (ρ = − 0.59)	NEU (ρ = − 0.43)	CRP (ρ = − 0.64)	CUPRAC (ρ = 0.67)
BChE	ALT (ρ = 0.38)						
TEAC	LVIDDn (ρ = − 0.40)	BUN (ρ = − 0.41)	Hp (ρ = − 0.49)	CUPRAC (ρ = 0.87)	thiol (ρ = 0.90)	PON1 (ρ = 0.60)	
CUPRAC	LVIDDn (ρ = − 0.52)	ALT (ρ = 0.38)	CRP (ρ = − 0.51)	Hp (ρ = − 0.53)			
Thiol	LVIDDn (ρ = −0.47)	ALT (ρ = 0.38)	BUN (ρ = − 0.41)	Cr (ρ = − 0.38)	CRP (ρ = − 0.43)	Hp (ρ = − 0.50)	CUPRAC (ρ = 0.89)
IL-2	ALT (ρ = − 0.45)	BChE (ρ = − 0.44)	GM-CSF (ρ = 0.70)				
IL-6	GM-CSF (ρ = 0.57)	IL-2 (ρ = 0.65)					
IL-7	LA/Ao (ρ = − 0.44)	ALT (ρ = − 0.37)	BChE (ρ = − 0.38)	GM-CSF (ρ = 0.68)	IL-2 (ρ = 0.68)	IL-6 (ρ = 0.49)	
IL-10	IFNg (ρ = 0.49)	KC-like (ρ = 0.41)					
IL-15	ALT (ρ = − 0.39)	GM-CSF (ρ = 0.85)	IL-2 (ρ = 0.78)	IL-6 (ρ = 0.70)	IL-7 (ρ = 0.67)		
IL-18	GM-CSF (ρ = 0.80)	IL-2 (ρ = 0.71)	IL-6 (ρ = 0.72)	IL-7 (ρ = 0.71)	IL-15 (ρ = 0.81)		
IP-10	Cr (ρ = 0.54)						
MCP-1	cTnI (ρ = 0.45)	LVIDDn (ρ = 0.41)	BUN (ρ = 0.39)	Hp (ρ = 0.40)	Ferritin (ρ = 0.38)	IL-6 (ρ = 0.66)	
GM-CSF	LA/Ao (ρ = − 0.44)	BChE (ρ = − 0.41)					
KC-like	cTnI (ρ = 0.45)	LA/Ao (ρ = 0.52)	WBC (ρ = 0.59)	NEU (ρ = 0.58)	CRP (ρ = 0.57)	Hp (ρ = 0.43)	Ferritin (ρ = 0.56)
TNF-α	GM-CSF (ρ = 0.65)	IL-2 (ρ = 0.85)	IL-6 (ρ = 0.73)	IL-7 (ρ = 0.71)	IL-15 (ρ = 0.76)	IL-18 (ρ = 0.78)	

## Discussion

To the best of the authors’ knowledge, this is the first study that reports changes in a panel of inflammatory and oxidative stress biomarkers in dogs with different stages of heart failure (stage A, B2, C and D) and their correlations with echocardiographic findings. In this study, CHF due to MMVD or DCM was diagnosed based on a thorough cardiopulmonary assessment as reported in the previous studies [5, 13], and classified by stage A through D according to the ACVIM consensus statement guidelines [3].

Accumulating evidence indicates that acute-phase proteins (APPs), inflammatory cytokines and oxidative stress may have a role in the pathogenesis of CHF in humans [19] and dogs [8, 20], but it is not known how inflammatory biomarkers changes in the different stages of heart failure. In this study, KC-like, which is an inflammatory chemokine, increased in serum of dogs with more severe stages. In addition increased concentrations of CRP and Hp and decreased activity of PON1 in stage D dogs showed that APPs may be associated with end-stage CHF. This would indicate that there is an inflammation associated with CHF specially in severe cases. Similar to our results, significantly higher serum CRP concentration was found in dogs with decompensated CHF compared with compensated dogs with heart disease and healthy dogs [13].

 The decrease in PON1 in dogs at stage D found in our study could indicate that the lack of the protective effect of PON1 could be involved in the more severe stages of CHF and is in agreement with the negative correlation between PON1 activity and severity of heart failure [21, 22]. PON1 is an enzyme with cardioprotective action in atherosclerosis and related vascular diseases [23], and its activity decreased in humans with CHF [24, 25] and also in situations of increased systemic oxidative stress and risk for cardiovascular disease such as diabetes mellitus and hypercholesterolemia in mouse [26] and humans [27]. Serum PON1 activity was suggested to be inversely associated with oxidative stress in serum and macrophages, with PON1 deficiency resulting in increased oxidative stress [27] and formation of reactive oxygen species (ROS) [21]. Therefore, the observed changes in chemokines, APPs and PON1 activity in this study provide further support for a role of systemic inflammatory activity and oxidative processes in the progression of CHF and a potential antioxidant compensatory role of PON1. In the present study, serum PON1 activity of dogs with DCM showed lower values than that of dogs with MMVD, in agreement with the study of Mahadesh-Prasad et al. [22] reporting a negative correlation of PON1 activity and severity of DCM.

Several reports have demonstrated enhanced expression and release of inflammatory cytokines and several chemokines in humans with CHF [28–30]. In this study, a panel of serum cytokines and chemokines was evaluated, and only serum KC-like levels were increased in symptomatic stages of CHF (stage C and D) compared to asymptomatic (stage B2) and healthy dogs (stage A). In a study similar to ours, Zois et al. [8] reported that MCP-1 chemokine was increased in CHF dogs compared to healthy dogs and some ILs decreased with disease severity. Information about KC-like, a major neutrophil chemoattractant, is limited, but it appears to play a role in systemic or generalized inflammation [31]. Increased plasma levels of KC-like have been associated with severe cardiac depression in old mice [12]. The observed positive correlations between KC-like and other analytes in our study (CRP, Hp, ferritin, and WBC and neutrophil counts) suggest an inflammatory role for KC-like. Inflammatory mediators may be released from the failing myocardium itself, and also from circulating WBC, platelets, endothelial cells, and from the liver and lungs and may contribute to myocardial depression and detrimental consequences such as endothelial dysfunction and cardiac myocyte apoptosis [32, 33].

In our study, lower values of all antioxidant biomarkers were found in severe stages of CHF. Similar to our findings, some of the antioxidant biomarkers such as thiol was decreased in humans with CHF [17]. Decreased myocardial contractility or pressure/volume overload leads to myocardial ischemia, which in turn induces a decrease in antioxidant compounds [34, 35] a. In the presented study, decreasing antioxidant capacity and antioxidant enzyme levels in serum of dogs with stage D may be due to ongoing oxidative stress during CHF that can influence the initiation and progression of valve lesions [25, 36, 37]. The decrease in antioxidant biomarkers could be connected with the increase in inflammatory biomarkers since the imbalance between antioxidants and oxidants can lead to induction of inflammatory cytokines [36, 38]. As changes in markers of inflammation and oxidative stress were more pronounced in stage D dogs, it can be suggested that the degree of inflammatory activity and antioxidant system impairment may be linked to the severity of the disease.

When the individual animals were evaluated before and after the treatment, there was in general, a decrease in the biomarkers of inflammation and an increase in the antioxidant biomarkers, which was associated with the clinical improvement of the dogs. Although the number of animals was low and these findings should be demonstrated in larger and more diverse groups of dogs with severe stages of HF, it could be postulated that biomarkers such as CRP and antioxidant biomarkers could have potential diagnostic and prognostic relevance to monitoring treatment in cases of severe CHF (stage D).

Our findings of a positive correlation between CRP levels and LV diameter and LA/Ao ratio are in agreement with a previous report made in dogs with CHF [6]. This finding and the negative correlation found between these cardiac variables and serum PON1 activity would indicate that APPs have possible role in the pathophysiology of CHF in dogs. MCP-1 protein was found to be correlated positively with cTnI and LVIDDn, suggesting that it may have an important role in myocardial damage and cardiac remodeling, as reported in human patients [39]. On the other hand, the negative correlation between serum CUPRAC, TEAC, and thiol levels and LV dimensions may indicate the role of antioxidant system deficit in the development of cardiac remodeling from stage B2 to stage D CHF. Studies have shown that deficit of antioxidant capacity may have a role for myocardial injury and then developing CHF [40, 41]. Based on these findings it could be postulated that antioxidant therapy or supplementation may be beneficial to slow or prevent the progression of CHF in dogs.

The present study is associated with several limitations. First, the sample size was small and dogs were not sub-divided as MMVD and DCM in this study, although this approach has been used in previous studies of changes in selected analytes in dogs with CHF [13, 42]. Therefore, this should be considered a pilot study and additional studies should be continued with a large number of animals with the same disease (MMVD or DCM or other diseases that can produce CHF) in separate groups. Second, there were wide-ranging variations in the body weight and various breeds of the included dogs. Both of them can influence several echocardiographic parameters such as LA and LV dimensions, LVIDDn, and EPSS. Although it showed no significant differences, the group had different body mass that could influence LVIDd, therefore the results of correlation between LVIDd and inflammatory and oxidative stress markers in addition to the hearth damage could have been influenced by the body mass changes. The LA/Ao ratio indicates the degree of LA dilation and shows a positive correlation with the severity of heart failure [43, 44]. LVIDDn is suggested as a more favorable indicator for evaluation of the degree of LV dilation in dogs [45]. Therefore, in this study, LA/Ao ratio and LVIDDn were used to describe echocardiographic evidence of cardiac remodeling. Third, ages in the control group should have been matched with the ages of the patient groups. Nevertheless, in the present study, mean ages in selected dogs were 3.5 yrs. (healthy control), 6.0 yrs. (stage B2), 8.6 yrs. (stage C), and 9.1 yrs. (stage D), in agreement with those of Reimann et al. [15]. In addition, gender and body weight were not associated with differences in concentrations of any cytokine [8].

## Conclusion

In conclusion, inflammatory markers such as CRP and KC-like are increased whereas antioxidant biomarkers such as TEAC, CUPRAC, and thiol are decreased in more severe stages of CHF, being these analytes correlated with some echocardiographic measurements. Some of these biomarkers of inflammation and oxidative stress could have the potential to be biomarkers to monitor the severity of the disease and the effect of treatment. Therapeutic strategies for preventing inflammation and oxidative stress may contribute to clinical improvement and slow disease progression.

## Methods

This study was performed between July 2018 and May 2019 at the Veterinary Teaching Hospital, Bursa Uludag University, Bursa / Turkey (Ethic ID: 2018–05 / 02).

### Dogs and groups

This study consisted of a total of 29 client-owned dogs of different breed, age, body weight, and both sexes. The dogs were classified according to the ACVIM staging system [3]. Dogs without evidence of cardiopulmonary and other diseases were included as healthy controls (stage A, *n* = 8). Stage B includes two subgroups (B1 and B2); asymptomatic and presence of heart murmur with (B2) or without cardiomegaly (B1). In this study, only B2 dogs (*n* = 6) were selected, which was characterized by the presence of heart murmur at mitral valve puncta maxima, and radiographic (vertebral heart score [VHS] > 10.5) and echocardiographic evidence (left atrial to aortic root ratio [LA/Ao] > 1.6 and/or body weight normalized left ventricular internal diameter in diastole [LVIDDN] > 1.7) of left-sided cardiac remodeling due to MMVD (*n* = 3) or DCM (*n* = 3). Stage C and D dogs were characterized by the presence of clinical signs associated with CHF. Stage C (*n* = 10) had a systolic heart murmur (≥ grade 3/6) over the mitral valve area with clinical (coughing, exercise intolerance, etc.), radiological (VHS > 11.0 and pulmonary edema) and echocardiographical evidence of left-sided cardiac remodeling as mentioned above due to MMVD (*n* = 7) or DCM (*n* = 3). Stage D dogs (*n* = 5) had a systolic heart murmur (grade 5–6/6), precordial thrill over the mitral valve area and abdominal distention (ascites), and echocardiographic evidence of left- and right-sided cardiac remodeling due to MMVD (*n* = 2) or DCM (*n* = 3), in addition to radiographic evidence of cardiomegaly (VHS > 11.5).

Just after the diagnosis of the disease, pimobendan (0.25 mg/kg, twice a day, PO) was prescribed alone for stage B2 patients, as suggested in EPIC study [46], or in combination with other medications; furosemide (2 mg/kg, once or twice a day, PO), spironolactone (2 mg/kg, once a day, PO), enalapril (0.5 mg/kg, once or twice a day, PO), and/or digoxin (0.005 mg/kg, twice a day, PO) for stage C patients [3]. Stage D CHF or advanced heart failure was defined as recurrence of CHF signs despite receiving the initially prescribed doses of standard medications with furosemide > 4 mg/kg/day. For these dogs, CHF treatment was revised as; pimobendan (0.3–05 mg/kg, twice daily, PO), torsemide (0.2 mg/kg, once or twice a day, PO), spironolactone (2 mg/kg, once a day, PO), enalapril in combination with thiazide diuretic (0.5 mg/kg, twice a day, PO), and if needed, anti-arrhythmic digoxin (0.005 mg/kg, twice a day, PO) and/or diltiazem (1 mg/kg, three times a day, PO) [47]. Two weeks later after revising of the medical therapy, dogs in stage D were re-examined to collect the data, for two-group comparison: pre- and post-treatment groups.

### Case selection

The diagnosis of MMVD was based on the combination of following criteria: the presence of mitral valve prolapse (MVP) and/or thickening of the mitral valve leaflets by 2-D echocardiography on right parasternal long-axis view, and identification of mitral valve regurgitation on left apical 4-chamber view by color Doppler examination [48, 49].

DCM was diagnosed based on the echocardiographic findings such as increased chamber size, increased E point to septal separation (EPSS) and poor fractional shortening (FS < 25%) along with ECG and thoracic radiographic findings. The diagnosis was confirmed using a scoring system (cut-off score: 6) for DCM proposed by the European Society for Veterinary Cardiology [5, 50]. If these dogs did not show the clinical signs, they were defined as a pre-clinical DCM (non-overt / asymptomatic DCM), and were included in stage B2 group. Overt or clinical DCM was diagnosed when the dogs had a total score above six, and showed the clinical signs such exercise intolerance, tachypnea, and coughing; according to treatment response, these dogs were included in stage C (good response) or stage D (refractory heart failure).

Healthy dogs were recruited from staff and students at the Veterinary Teaching Hospital. All dogs were healthy based on normal physical and cardiovascular examinations and laboratory assessments in which the results of complete blood count (CBC), serum cardiac troponin I (cTnI) and serum biochemistry profile were within the reference ranges suggested for dogs.

### Exclusion criteria

According to the results of the analysis, dog with comorbidities such as infectious diseases (pneumonia, urinary tract disease or pyoderma, etc.), non-infectious diseases (renal failure, atopy, inflammatory bowel disease or hepatitis), vector-borne diseases (ehrlichiosis, Lyme, and dirofilariasis, etc.), endocrine diseases (diabetes mellitus, hypothyroidism, hyperthyroidism and Cushing disease, among others) and patients with benign or malignant tumors were excluded. If the dogs received any kind of medication (steroids, non-steroids, antibiotics, inotropes or diuretics, etc) prior to admission to the clinic, they were not included to the study, because of the fact that some medication could affect on hematologic and serum biochemistry profile which were analysed here.

## Sample collection and measurements

### Examinations of the cardiopulmonary system

In this study, the cardiopulmonary system was evaluated by a thorough physical examination, electrocardiography (ECG), thoracic radiography and echocardiography in all dogs. Physical examination included body temperature, heart and respiratory rates and cardiac auscultation etc. Bilateral, ventrodorsal and/or dorsoventral radiographs of each patient were taken, and radiological morphology of the heart, vertebral heart score, lung and thoracic vessels were examined. ECG was recorded without sedation using 3 bipolar standard limb leads. Cardiac rhythm analyses and measurements were performed with a standard calibration (10 mm/mV and 50 mm/sec), as reported in a previous study [51].

A transthoracic echocardiographic examination was performed as reported previously [13, 51, 52]. Briefly, cardiac measurements were done using conventional modalities (2-D, M-mode, and color Doppler) and imaging techniques (right parasternal short and long axis, left apical 4–5 chamber and subcostal views) with phased-array cardiac transducers in all dogs (Caris Plus Esaote, Italy). Left ventricular related parameters such as left ventricular internal diameter at diastole (LVIDd) and systole (LVIDs) were calculated by the Teichholz method derived from M-mode measurements, at right parasternal long axis view.

### Laboratory analysis

Venous blood samples were collected via venipuncture from the brachiocephalic veins into EDTA tubes for CBC and serum tubes for biochemistry (inflammatory biomarkers, cytokine panel, and oxidative stress markers) and cardiac troponin I (cTnI) analyses. Serum samples were stored at − 80 °C for a maximum of 8 months until analysis.

### Hematological and serum biochemistry analysis

CBC was measured in the animal hospital lab within 1 h after blood collection (HM5, Abaxis), and only white blood cell (WBC) and neutrophil counts were presented in this study. In all dogs, routine serum biochemistry panel including enzyme activities (ALP, ALT, CK, and amylase), total protein, electrolytes (Ca, P), renal damage markers (blood urea nitrogen and creatinine), glucose and total bilirubin was measured (Comprehensive Diagnostic Profile Rotor, VetScan, Abaxis). Serum thyroxine and cholesterol levels were measured using T4/Cholesterol Reagent Rotor (VetScan, Abaxis). Serum cTnI was measured with a portable clinical device (cTnI cartridge, I-Stat, Abaxis).

### Inflammatory biomarkers

Serum ferritin concentration was measured using a commercial immunoturbidimetric assay (Tina-quant Ferritin, Roche). A commercially available method (Tridelta Ltd., Brey, Ireland) was used for haptoglobin (Hp) concentration measurement. C-reactive protein (CRP) was measured in serum using an immunoturbidimetric assay (CRP OSR6147 Olympus Life and Material Science Europe GmbH, Hamburg, Germany). PON1 and BChE activities were determined following previously validated assays [53, 54]. All analysis was performed using the Olympus AU600 (Olympus Diagnostica GmbH) analyzer.

### Serum cytokines measurements

Milliplex® MAP magnetic bead panel (CCYTO-90 K Millipore, Billerica, MA) with an automated analyzer (Luminex 200, Luminex Corporation, Austin, TX) was used to determine concentrations of 13 cytokines (interleukin-2 (IL-2), IL-6, IL-7, IL-8, IL-10, IL-15, IL-18, interferon gamma-induced protein 10 (IP-10), monocyte chemoattractant protein 1 (MCP-1), granulocyte-macrophage colony-stimulating factor (GM-CSF), keratinocyte-derived chemokine (KC)-like, tumour necrosis factor-alpha (TNF-α) and interferon-gamma (IFN-γ) in blood serum. The assay was performed according to the manufacturer’s instructions. Internal quality control material provided by the manufacturer was used to generate a standard curve and calculate concentration for each analyte.

### Oxidative stress biomarkers

Trolox equivalent antioxidant capacity (TEAC), based on the enzymatic generation of ABTS radical, and cupric reducing antioxidant capacity (CUPRAC) were determined in serum by using previously validated assays [55, 56]. Serum thiol concentrations were measured using the method described by Jocelyn [57] and modified by Costa et al. [58].

### Statistical analysis

Data were analyzed using a commercial software tool (GraphPad Prism 6, San Diego, USA). Changes in results between the different groups were assessed by a non-parametric test (Kruskal–Wallis followed by Dunn’s multiple comparison) because of the small sample size. Therefore, they were presented as median and interquartile range. Correlations between variables were determined using the Spearman test. A *P* < 0.05 was taken as statistically significant in all cases. Figures were produced with different colors representing different disease; DCM (red color) and MMVD (blue color).

## Data Availability

All data in this study will be available from the corresponding author upon reasonable previous request and with the permission of the research fund.

## Abbreviations

2-D: Two dimensional
ACVIM: American College of Veterinary Internal Medicine
ALP: Alkaline phosphatase
ALT: Alanine aminotransferase
APPs: Acute-phase proteins
BChE: Butyrylcholinesterase
CBC: Complete blood count
CHF: Chronic heart failure
CK: Creatinine kinase
CRP: C-reactive protein
cTnI: Cardiac troponin I
CUPRAC: Cupric reducing antioxidant capacity
DCM: Dilated cardiomyopathy
ECG: Electrocardiography
EJ: Ejection fraction
EPSS: E-point to septal separation
FS: Fractional shortening
GM-CSF: Granulocyte macrophage-colony stimulating factor
Hp: Haptoglobin
IFN-γ: Interferon gamma
IL: Interleukin
IP-10: Interferon γ-induced protein 10
KC-like: Keratinocyte chemoattractant-like
LA/Ao: Left atrium to aorta ratio
LV: Left ventricle
LVDd: Left ventricular diastole diameter
LVIDDn: Normalized left ventricular internal diameter in diastole
MCP-1: Monocyte chemoattractant protein 1
MMVD: Myxomatous mitral valve degeneration
MVP: Mitral valve prolapse
PLT: Platelet
PON-1: Paraoxonase 1
RBC: Red blood cell
ROS: Reactive oxygen species
T4: Thyroxine
TEAC: Trolox equivalent antioxidant capacity
TNF-α: Tumour necrosis factor
VHS: Vertebral heart score
WBC: White blood cell

## References

[1] Ribeiro-Samora GA, Rabelo LA, Ferreira ACC, Favero M, Guedes GS, Pereira LSM, Parreira VF, Britto RR (2017). Inflammation and oxidative stress in heart failure: effects of exercise intensity and duration. Braz J Med Biol Res.

[2] Janus I, Kandefer-Gola M, Ciaputa R, Noszczyk-Nowak A, Pasławska U, Tursi M, Nowak M (2016). The immunohistochemical evaluation of selected markers in the left atrium of dogs with end-stage dilated cardiomyopathy and myxomatous mitral valve disease - a preliminary study. Ir Vet J.

[3] Keene BW, Atkins CE, Bonagura JD, Fox PR, Häggström J, Fuentes VL, Oyama MA, Rush JE, Stepien R, Uechi M (2019). ACVIM consensus guidelines for the diagnosis and treatment of myxomatous mitral valve disease in dogs. J Vet Intern Med.

[4] Simpson S, Edwards J, Ferguson-Mignan TF, Cobb M, Mongan NP, Rutland CS. Genetics of human and canine dilated cardiomyopathy. Int J Genomics. 2015:204823. 10.1155/2015/204823. 10.1155/2015/204823 PMC4525455 26266250

[5] Borgarelli M, Santilli RA, Chiavegato D, Agnolo GD, Zanatta R, Mannelli A, Tarducci A (2006). Prognostic indicators for dogs with dilated cardiomyopathy. J Vet Intern Med.

[6] Reimann MJ, Ljungvall I, Hillström A, Møller JE, Hagman R, Falk T, Höglund K, Häggström J, Olsen LH (2016). Increased serum C-reactive protein concentrations in dogs with congestive heart failure due to myxomatous mitral valve disease. Vet J.

[7] Schultheiss H, Fairweather D, Caforio ALP, Escher F, Hersheberger RE, Lipshultz SE, Liu PP, Matsumori A, Mazzanti A, McMurray J, Priori SG (2019). Dilated cardiomyopathy. Nat Rev Dis Primers.

[8] Zois NE, Moesgaard SG, Kjelgaard-Hansen M, Rasmussen CE, Falk T, Fossing C, Häggström J, Pedersen HD, Olsen LH (2012). Circulating cytokine concentrations in dogs with different degrees of myxomatous mitral valve disease. Vet J.

[9] Fonfara S, Hetzel U, Tew SR, Cripps P, Dukes-McEwan J, Clegg PD (2013). Myocardial cytokine expression in dogs with systemic and naturally occurring cardiac diseases. Am J Vet Res.

[10] Vatnikov Y, Rudenko A, Rudenko P, Kulikov E, Karamyan A, Lutsay V, Medvedev I, Byakhova V, Krotova E, Molvhanova M (2019). Immune-inflammatory concept of the pathogenesis of chronic heart failure in dogs with dilated cardiomyopathy. Vet World.

[11] Dekker RL, Moser DK, Tovar EG, Chung ML, Heo S, Wu JR, Dunbar SB, Pressler SJ, Lennie TA (2014). Depressive symptoms and inflammatory biomarkers in patients with heart failure. Eur J Cardiovasc Nurs.

[12] Slimani H, Zhai Y, Yousif NG, Ao L, Zeng Q, Fullerton DA, Meng X. Enhanced monocyte chemoattractant protein-1 production in aging mice exaggerates cardiac depression during endotoxemia. Crit Care. 2014;18. 10.1186/s13054-014-0527-8. 10.1186/s13054-014-0527-8 PMC4172828 25209241

[13] Domanjko PA, Lukman T, Verk B, Nemec SA (2018). Systemic inflammation in dogs with advanced - stage heart failure. Acta Vet Scand.

[14] Freeman LM, Rush JE, Milbury PE, Blumberg JB (2009). Antioxidant status and biomarkers of oxidative stress in dogs with congestive heart failure. J Vet Intern Med.

[15] Reimann MJ, Häggström J, Møller JE, Lykkesfeldt J, Falk T, Olsen LH (2017). Markers of oxidative stress in dogs with myxomatous mitral valve disease are influenced by sex, neuter status, and serum cholesterol concentration. J Vet Intern Med.

[16] Verk B, Nemec Svete A, Salobir J, Rezar V, Domanjko Petrič A (2017). Markers of oxidative stress in dogs with heart failure. J Vet Diagn Investig.

[17] Belch JJF, Bridges AB, Scott N, Chopra M (1991). Oxygen free radicals and congestive heart failure. Br Heart J.

[18] Díaz-Vélez CR, García-Castiñeiras S, Mendoza-Ramos E, Hernández-López E (1996). Increased malondialdehyde in peripheral blood of patients with congestive heart failure. Am Heart J.

[19] Deswal A, Petersen NJ, Feldman AM, Young JB, White BG, Mann DL (2001). Cytokines and cytokine receptors in advanced heart failure: an analysis of the cytokine database from the Vesnarinone trial (VEST). Circulation.

[20] Polizopoulou ZS, Koutinas CK, Cerón JJ, Tvarijonaviciute A, Martínez-Subiela S, Dasopoulou A, York MJ, Roman IF, Gandhi M, Patel S, O'Brien PJ (2015). Correlation of serum cardiac troponin I and acute phase protein concentrations with clinical staging in dogs with degenerative mitral valve disease. Vet Clin Pathol.

[21] Kulka M, Garncarz M, Parzeniecka-Jaworska M, Kluciński W (2017). Serum paraoxonase 1 activity and lipid metabolism parameter changes in Dachshunds with chronic mitral valve disease. Assessment of its diagnostic usefulness. Pol J Vet Sci.

[22] Mahadesh Prasad AJ, Krueger M, Krueger M (2014). Decreased level of serum paraoxonase (PON) activity in dogs with dilated cardiomyopathy (DCM). J Vet Med Anim Health.

[23] Chistiakov DA, Melnichenko AA, Orekhov AN, Bobryshev YV. Paraoxonase and atherosclerosis - related cardiovascular diseases. Biochimie. 2017. 10.1016/j.biochi.2016.10.010. 10.1016/j.biochi.2016.10.010 27771368

[24] Kim JB, Hama S, Hough G, Navab M, Fogelman AM, Maclellan WR, Horwich TB, Fonarow GC (2013). Heart failure is associated with impaired anti-inflammatory and antioxidant properties of high-density lipoproteins. Am J Cardiol.

[25] Eren E, Ellidağ HY, Aydin O, Küçükseymen S, Giray O, Aslan S, Yılmaz N (2015). The relationship between HDL-associated PON1 activity, oxidative stressand brain natriuretic peptide in NYHA functional class heart failure patients. Biomed Res.

[26] Shih DM, Lusis AJ (2009). The roles of PON1 and PON2 in cardiovascular disease and innate immunity. Curr Opin Lipidol.

[27] Elkiran ET, Mar N, Aygen B, Gursu F, Karaoglu A, Koca S (2007). Serum paraoxonase and arylesterase activities in patients with lung cancer in a Turkish population. BMC Cancer.

[28] Aukrust P, Ueland T, Muller F, Andreassen AK, Aass H, Kjekshus J, Simonsen S, Frøland SS, Gullestad L (1998). Elevated circulating levels of C-C chemokines in patients with congestive heart failure. Circulation.

[29] Aukrust P, Ueland T, Lien E, Bendtzen K, Muller F, Andreassen AK, Nordøy I, Aass H, Espevik T, Simonsen S, Frøland SS, Gullestad L (1999). Cytokine network in congestive heart failure secondary to ischemic or idiopathic dilated cardiomyopathy. Am J Cardiol.

[30] Gullestad L, Ueland T, Vinge L, Finsen A, Yndestad A, Aukrust P (2012). Inflammatory cytokines in heart failure: mediators and markers. Cardiology.

[31] Huo Y, Weber C, Forlow SB, Sperandio M, Thatte J, Mack M, Ley K (2001). The chemokine KC, but not monocyte chemoattractant protein-1, triggers monocyte arrest on early atherosclerotic endothelium. J Clin Invest.

[32] Yndestad A, Damås JK, Øie E, Ueland T, Gullestad L, Aukrust P. Systemic inflammation in heart failure - the whys and wherefores. Heart Fail Rev. 2006. 10.1007/s10741-006-9196-2. 10.1007/s10741-006-9196-2 16819581

[33] Cunningham SM, Rush JE, Freeman LM (2012). Systemic inflammation and endothelial dysfunction in dogs with congestive heart failure. J Vet Intern Med.

[34] Guarnieri C, Flamigni F, Caldarera CM (1980). Role of oxygen in the cellular damage induced re-oxygenation of hypoxic heart. J Mol Cell Cardiol.

[35] Michałek M, Tabiś A, Cepiel A, Noszczyk-Nowak A (2020). Antioxidative enzyme activity and total antioxidant capacity in serum of dogs with degenerative mitral valve disease. Can J Vet Res.

[36] Mak S, Newton GE (2001). The oxidative stress hypothesis of congestive heart failure: radical thoughts. Chest.

[37] Tanaka R, Shimizu M. The relationship between reactive oxygen species and cardiac fibrosis in the Dahl salt-sensitive rat under ACEI administration. Vet Med Int. 2012:105316. 10.1155/2012/105316. 10.1155/2012/105316 PMC3329713 22577606

[38] Prasad K, Gupta JB, Kalra J, Lee P, Mantha SV, Bharadwaj B (1996). Oxidative stress as a mechanism of cardiac failure in chronic volume overload in canine model. J Mol Cell Cardiol.

[39] Ritter A, Faria A, Sabbatini A, Corrêa NB, Brunelli V, Modolo R, Moreno H (2017). MCP-1 levels are associated with cardiac remodeling but not with resistant hypertension. Arq Bras Cardiol.

[40] Hill MF, Singal PK (1996). Antioxidant and oxidative stress changes during heart failure subsequent to myocardial infarction in rats. Am J Pathol.

[41] van der Pol A, van Gilst WH, Voors AA, van der Meer P (2019). Treating oxidative stress in heart failure: past, present and future. Eur J Heart Fail.

[42] Damoiseaux C, Merveille AC, Krafft E, Da Costa AM, Gomart S, Jespers P, Michaux C, Clercx C, Verhoeven C, Mc Entee K (2014). Effect of physiological determinants and cardiac disease on plasma adiponectin concentrations in dogs. J Vet Intern Med.

[43] Han D, Lee DG, Jung DI (2016). Echocardiographic evaluation of heart failure in dogs with myxomatous mitral valve disease: a retrospective study. J Biomed Transl Res.

[44] Hansson K, Haggstrom J, Kvart C, Lord P (2002). Left atrial to aortic root indices using two-dimensional and M-mode echocardiography in Cavalier King Charles Spaniels with and without left atrial enlargement. Vet Radiol Ultrasound.

[45] Cornell CC, Kittleson MD, Della Torre P, Haggstrom J, Lombard CW, Pedersen HD, Vollmar A, Wey A (2004). Allometric scaling of M-mode cardiac measurements in normal adult dogs. J Vet Intern Med.

[46] Boswood A, Häggström J, Gordon SG (2016). Effect of Pimobendan in dogs with preclinical myxomatous mitral valve disease and cardiomegaly: the EPIC study-a randomized clinical trial. J Vet Intern Med.

[47] Beaumier A, Rush JE, Yang VK, Freeman LM (2018). Clinical findings and survival time in dogs with advanced heart failure. J Vet Intern Med.

[48] Häggström J, Duelund Pedersen H, Kvart C (2004). New insights into degenerative mitral valve disease in dogs. Vet Clin North Am Small Anim Pract.

[49] Höllmer M, Willesen JL, Tolver A, Koch J (2017). Left atrial volume and function in dogs with naturally occurring myxomatous mitral valve disease. J Vet Cardiol.

[50] Dukes-McEwan J, Borgarelli M, Tidholm A, Vollmar AC, Häggström J, ESVC Taskforce for Canine Dilated Cardiomyopathy (2003). Proposed guidelines for the diagnosis of canine idiopathic dilated cardiomyopathy. J Vet Cardiol.

[51] Kocaturk M, Salci H, Yilmaz Z, Bayram AS, Koch J (2010). Pre- and post-operative cardiac evaluation of dogs undergoing lobectomy and pneumonectomy. J Vet Sci.

[52] Kocaturk M, Martinez S, Eralp O, Tvarijonaviciute A, Ceron J, Yilmaz Z (2012). Tei index (myocardial performance index) and cardiac biomarkers in dogs with parvoviral enteritis. Res Vet Sci.

[53] Tecles F, Martínez-Subiela S, Bernal LJ, Cerón JJ (2000). Use of whole blood for spectrophotometric determination of cholinesterase activity in dogs. Vet J.

[54] Tvarijonaviciute A, Kocaturk M, Cansev M, Tecles F, Ceron JJ, Yilmaz Z (2012). Serum butyrylcholinesterase and paraoxonase 1 in a canine model of endotoxemia: effects of choline administration. Res Vet Sci.

[55] Rubio CP, Hernández-Ruiz J, Martinez-Subiela S, Tvarijonaviciute A, Arnao MB, Ceron JJ (2016). Validation of three automated assays for total antioxidant capacity determination in canine serum samples. J Vet Diagn Investig.

[56] Rubio CP, Tvarijonaviciute A, Martinez-Subiela S, Hernández-Ruiz J, Ceron JJ (2016). Validation of an automated assay for the measurement of cupric reducing antioxidant capacity in serum of dogs. BMC Vet Res.

[57] Jocelyn P (1987). Spectrophotometric assay of thiols. Methods Enzymol.

[58] Costa CM, da Santos RCC, dos Lima ES (2006). A simple automated procedure for thiol measurement in human serum samples. J Bras Patol eMed Lab.

